# Influence of Laser Metal Deposition Process Parameters on the Structural Integrity of CuSn_11_Bi_3_ Coatings on C45

**DOI:** 10.3390/ma18184368

**Published:** 2025-09-18

**Authors:** Federico Mazzucato, Edouard Baer, Samuel Rey-Mermet, Anna Valente

**Affiliations:** 1 Department of Innovative Technologies, University of Applied Science and Arts of Southern Switzerland (SUPSI), Via La Santa 1, 6962 Lugano, Switzerland; anna.valente@supsi.ch; 2 Institute of Systems Engineering, School of Engineering, University of Applied Sciences and Arts HES-SO Valais-Wallis, Systems Engineering Rue de l’Industrie 23, 1950 Sion, Switzerland; edouard.baer@hevs.ch (E.B.); samuel.rey-mermet@hevs.ch (S.R.-M.)

**Keywords:** Laser Metal Deposition, Cu-based alloys, multi-material, chemical analysis, bimetallic structure

## Abstract

Bronze-steel bimetallic structures are structural components finding a growing application in industrial sectors such as aerospace, power generation, and machinery. Recent legislation on green economy and sustainable manufacturing is boosting industry to implement innovative manufacturing processes and new metal alloys capable of lowering environmental footprint by avoiding toxic substances. Laser Metal Deposition is a cost-effective Additive Manufacturing technique for producing bimetallic components by limiting material waste and reducing energy consumption. In this research work, the influence of the main LMD process parameters on the final quality of CuSn_11_Bi_3_ coatings on C45 surfaces is analyzed. The Cu-based powder is specifically designed and developed to reduce environmental pollution and increase worker safety by avoiding the use of hazardous chemical elements. The performed observations demonstrate that high-density (99.90%) and crack-free clads are feasible by preventing melt pool dilution zones. Cu diffusion into the C45 substrate deteriorates the structural integrity at the clad-substrate interface by inducing liquid metal embrittlement cracking, whereas steel diffusion into the as-deposited clad promotes crack propagation. High-density (up to 99.97%) and crack-free CuSn_11_Bi_3_ coatings are achieved by using low specific energies (from 17 J/mm^2^ to 40 J/mm^2^) and reducing the Oxygen content during sample manufacturing up to 0.02%.

## 1. Introduction

Bimetallic structures consisting of a steel bulk coated with pure or pre-alloyed copper (Cu) are advanced structural components that are finding growing applications in industrial sectors such as aerospace, machinery, and power generation [1,2,3]. Specifically, copper-steel composites are conceived to take advantage of the joining of the dissimilar thermal, chemical, and mechanical properties of their heterogeneous constituents, exhibiting unprecedented performance not achievable by mono-material solutions in terms of high mechanical strength, improved corrosion and cavitation resistance, low dynamic friction coefficient, and high thermal conductivity [4,5,6,7]. Examples of copper-steel bimetallic components are bearing bushes, cooling staves, liquid pipelines, and hydraulic pumps [8]. Currently, the primary production method of such bimetallic components consists of casting pure Cu or Cu-based alloys on a pre-machined steel bulk, followed by heat treatments and a final step of machining to meet product requirements in terms of mechanical and tribological properties, dimensional accuracy, geometrical tolerances, and surface roughness. Despite the above fabrication method proving to be reliable in the production of high-quality structural components, it exhibits several drawbacks in terms of limited achievable part geometrical complexity, low process repeatability, high material waste, and high CO_2_ emissions. Moreover, recent stringent legislations concerning circular economy and sustainable manufacturing (e.g., REACH Regulation [9]) are forcing the industry to renew its workshops by embracing cutting-edge manufacturing technologies capable of managing new metal alloys, ensuring high standards in the production of high-quality structural parts with reduced resource consumption and lower pollution and carbon emissions [10,11,12]. Direct Energy Deposition (DED) is a family of Additive Manufacturing (AM) techniques that has proven to be a cost-effective alternative to casting for the fabrication of both Functionally Graded Materials (FGMs) [13,14,15] and high-quality functional surface coatings [16,17,18,19] by decreasing the environmental impacts in terms of greenhouse gas emissions, scraps, and raw material management. Among DED processes, powder-based Laser Metal Deposition (LMD) is a recognized AM technique enabling an efficient optimization of the part design through an advanced functionalization of the material gradient structure [20]. Compared to Powder Bed Fusion (PBF), the LMD process fabricates metal parts layer upon layer by means of local deposition of metal powder injected into a melt pool, which is created by a constant interaction between a laser source and a metal surface [21]. When the laser moves, the melt pool solidifies, and a solid metal bonding between the as-deposited material and the underlying surface forms [22,23,24,25,26,27]. The capability to tailor the metal powder chemical composition on-the-fly and the limited Heat Affected Zone (HAZ) generated during the deposition process makes LMD a suitable and cost-effective manufacturing technique to realize high-quality graded structures [28,29,30,31].

Several authors have analyzed the influence of LMD process parameters on the manufacturing of defect-free Cu depositions. The scientific community agrees that the realization of high-density bulky features is feasible through LMD IR laser sources, but process optimization remains critical and resource-intensive due to the high reflectivity of the involved metal powder [32,33]. Moreover, an inert deposition chamber is required to prevent the deterioration of the tribological properties of the as-built Cu coating due to O_2_ solubilization, which forms Cu_2_O precipitates in the as-built microstructure [34]. Xiao et al. [35] were successful in repairing a motor commutator by depositing Cu-based metal powder (Cu_15_Sn) on a pure Cu surface via a blue laser. Compared to the base material, they achieved a defect-free clad formation exhibiting a 2.22 times higher microhardness and 2.37 times wear resistance under the testing conditions of dry friction and wear at room temperature, proving that the final quality of the realized metallic components and the efficiency of the LMD process increase when using Cu-based alloys instead of pure Cu or blue laser sources instead of IR. Despite blue lasers ensuring enhanced deposition efficiency thanks to a higher coefficient of energy absorption [36,37,38], their service life, beam quality performance over time, and reliability are still one step behind IR systems, making blue thermal sources not yet mature for industrial production.

When dealing with bronze-steel bimetallic composites, the LMD of Cu-based alloys becomes even more challenging. Differences in the coefficients of thermal expansion, disparity in the melting temperatures and cooling rates, diverse crystal structures, and embrittlement phenomena undermine the structural integrity of the realized bimetallic structure by introducing structural cracks and delamination [39,40]. Recent research has focused on preventing the formation of detrimental defects, such as solidification cracking along the as-deposited Cu grain boundaries and liquid metal embrittlement (LME) cracks, by deploying a metallic interlayer between the two dissimilar metals [41]. Zhang et al. [42] demonstrated that defect-free bronze-SS304L bonding is achieved through a Deloro 22 interlayer deposition. Moreover, Chen et al. [43] and Wang et al. [44] confirmed that Nickel has an inhibitory effect on the formation of LME cracks in the steel substrate when Cu-based alloys are deposited. Despite the above-mentioned advantages, the implementation of an interlayer is not always feasible at the industrial level due to the diffusion of undesired chemical elements (e.g., Ni) into the as-deposited clad, which affects both the tribological behavior, such as friction coefficients and wear/corrosion resistance, and the thermal properties (e.g., thermal conductivity) of the Cu-based coating [45].

The objective of this research work is to characterize the influence of the powder-based LMD process parameters on the final quality of CuSn_11_Bi_3_ depositions performed on C45 surfaces to enable high-quality superficial coatings without the need for interlayers. The Cu-based powder used in this experimental activity is a new lead-free bronze alloy designed and developed to comply with the REACH regulation by ensuring high mechanical performance and enhanced tribological properties for hydraulic industrial applications [46]. The influence of laser power, axis speed, and plate preheating on the internal and chemical quality of as-built Cu-based clads is analyzed by assessing single track cross-sections. Acceptable combinations of LMD process parameters ensuring high-density and crack-free bronze depositions are used to realize single layers and assess the impact of the deposition environment on the final structural integrity of the performed coating. The achieved experimental results demonstrate that high-density CuSn_11_Bi_3_ depositions on C45 are achieved for low specific energies (i.e., ranging from 40 J/mm^2^ to 17 J/mm^2^), whereas keyhole phenomena and LME cracking occur at high laser power. Crack formation and propagation are avoided by preventing steel diffusion into the Cu-based clad and keeping the content of Oxygen (O_2_) during material deposition below 0.02%. Section 2 deals with the material and equipment involved in the experimental campaign execution and sample analysis. Section 3 presents and discusses the achieved results. Finally, Section 4 and Section 5, respectively, introduce the main conclusions and future works.

## 2. Materials and Methods

### 2.1. Metal Material and LMD Equipment

**Figure 1:**
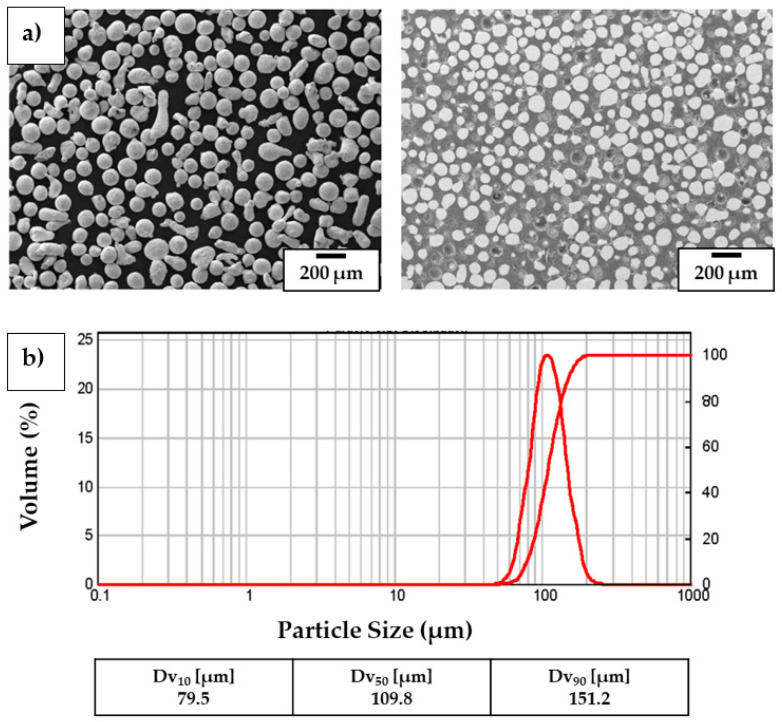
CuSn_11_Bi_3_ powder particles: (**a**) particle shape and cross-section; (**b**) particle grain size distribution.

The metal powder involved in the experimental campaign was a CuSn_11_Bi_3_ spherical-shaped powder [46] designed and developed by Kugler Bimetal SA (Le Lignon, Switzerland) for the manufacturing of advanced coatings for the hydraulic, power generation, and aerospace sectors. The powder grain size ranged from 79.5 µm to 151.2 µm, and powder tap density was 4.9 g/cm^3^ (see Figure 1).

The metal substrate was an 18 mm thick C45 steel disk (supplier: Brütsch/Rüegger Metals AG (Regensdorf, Switzerland)) with a nominal diameter of 115 mm. The material coupling was based on an industrial use case concerning a core component of a hydraulic pump and consisted of a bulk of medium carbon steel coated with a Cu-based alloy to ensure high mechanical strength in conjunction with enhanced superficial tribological properties. Table 1 shows the chemical composition of both the CuSn_11_Bi_3_ powder and the C45 disk, whereas Table 2 shows the main thermo-mechanical properties of the two metallic materials.

**Figure 2:**
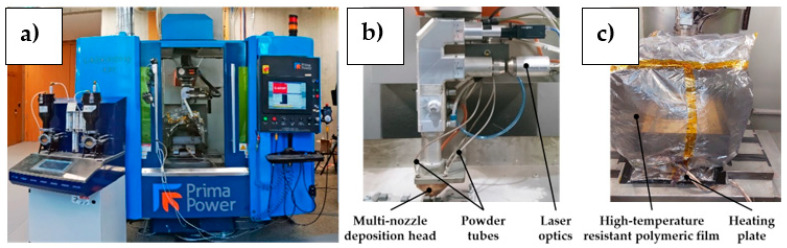
(**a**) Laserdyne 430 LMD system; (**b**) LMD deposition head; (**c**) enclosed deposition chamber.

The LMD system was a three-axis Laserdyne 430 machine, Prima Power, Turin, Italy, that consisted of a laser cutting machine retrofitted and adapted to perform the powder-based LMD process (see Figure 2a). The system was equipped with a Convergent Photonics CF1000 laser source, Convergent, France, featuring 1000 W maximum power, a nominal laser spot of 1 mm, and a wavelength of 1070 nm. A heating plate designed and developed by the Automation, Robotics, and Machines laboratory of SUPSI was implemented below the sample clamping system to enable a maximum preheating temperature of 450 °C on the substrate surface. Powder supply was ensured by a LENS^TM^ Print Engine double-hopper powder feeding system, Optomec, NM, USA, connected to a commercial Optomec^®^ multi-nozzle deposition head, Optomec, NM, USA (see Figure 2b). The inert gas used as a carrier and shielding gas was Argon 4.6. To reduce the O_2_ content during the deposition process, the building volume was enclosed by a high-temperature resistant polymeric film (see Figure 2c), and the deposition chamber was filled with Argon to reach an O_2_ content below 0.02% before executing CuSn_11_Bi_3_ depositions.

### 2.2. Experimental Campaign

**Figure 3:**
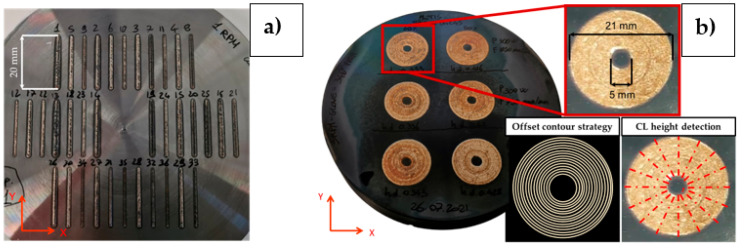
Performed CuSn_11_Bi_3_ depositions: (**a**) single tracks (STs); (**b**) circular layers (CLs).

The experimental campaign consisted of two main phases: (1) the deposition and characterization of linear CuSn_11_Bi_3_ tracks on C45 disks (see Figure 3a); the realization and assessment of bidimensional coatings produced using suitable combinations of LMD process parameters, ensuring high-quality linear CuSn_11_Bi_3_ depositions (see Figure 3b).

The influence of the laser power (P), axis speed (F), and substrate preheating on the final structural quality of 20 mm single linear CuSn_11_Bi_3_ depositions, i.e., single tracks (STs), was analyzed by taking six levels for P, six levels for F, and two levels of substrate preheating temperatures (see Table 3). The reason for choosing P ranging from 300 W to 800 W and F ranging from 300 mm/min to 1050 mm/min was for the following:

- Based on the results discussed in previous studies involving the same LMD system [18,47], using a P lower than 300 W or an F higher than 1050 mm/min resulted in unstable depositions featuring a lack of fusion.
- A maximum P level of 800 W was chosen based on Zhang et al. [48] and Kim et al. [49], who suggested not exceeding an energy density threshold of 80 J/mm^2^ to prevent keyhole formation. Given the high reflectivity of the Cu-based powder involved in this study and the corresponding energy dispersion, the authors decided to overcome the recommended threshold and set a maximum value of 160 J/mm^2^ to have a larger overview of the iteration behavior between LMD parameters and material.

**Figure 4:**
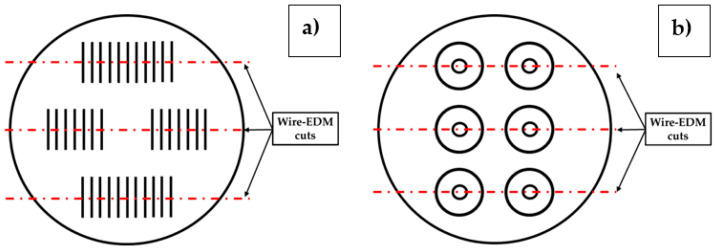
Performed EDM cut positions for (**a**) single tracks (STs); (**b**) circular layers (CLs).

Three repetitions for each combination of process parameters were performed for a total of 216 realized STs. To enable internal quality assessment, each realized sample was cut by wire-EDM (see Figure 4a), and the resulting cross-sections were pre-polished with SiC-coated paper (FEPA 120), polished with diamond particles of 6, 3, and 1 μm, and finished with Oxide Polishing Suspension (OPS). A Zeiss Gemini 460 Scanning Electron Microscope (SEM), Zeiss, Oberkochen, Germany, was used to characterize the structural integrity of the clad cross-sections in terms of pores, cracks, and delamination. Specifically, porosity measurements were performed using the open-source ImageJ software v1.54p, which was used to both compute the pore size and the percentage in the analyzed area. After acquiring the ST cross-section with the SEM, the image was opened in ImageJ, the pixels were calibrated based on the scale bar of the SEM image, and the pore size was measured. The porosity percentage was calculated by determining the fraction of area occupied by pores relative to the total using the “Analyse Particles” tool in the software. Data analysis and representation were performed through Minitab Statistical Software v22 and Excel. The material diffusion and final chemical composition of the realized samples were evaluated through Energy Dispersive X-ray (EDX) analysis performed using an EDX Ultimax 100 system, Oxford Instruments, United Kingdom.

The manufacturing of bidimensional coatings, i.e., Circular Layers (CLs), was implemented by using the combinations of process parameters resulting in crack-free and high-density CuSn_11_Bi_3_ linear clads (see Table 4). CL fabrication was performed with and without the implementation of a high-temperature resistant polymeric film (i.e., “enclosed” and “open” deposition chambers, ensuring an O_2_ content of 0.02% and 21%, respectively) to analyze the influence of two different environmental printing conditions on the structural and dimensional quality of the performed coatings. Figure 3b shows the nominal size of the realized coatings and the implemented offset contour deposition strategy by using 60% ST overlapping. As performed for the linear CuSn_11_Bi_3_ depositions, each combination of process parameters was repeated three times for a total of 36 realized CLs. The final quality of the realized CLs was assessed in terms of layer final height, computed by averaging 16 equally spaced CL profiles traced radially through a Keyence ^®^ VHX-6000 digital microscope, Keyence, Japan, that involved 1.47 µm planar resolution and 0.2 µm vertical pitch (see Figure 3b), and structural integrity by following the same methodology implemented for SL and performing the wire-EDM cuts shown in Figure 4b.

## 3. Results and Discussion

### 3.1. CuSn_11_Bi_3_ ST Analysis

**Figure 5:**
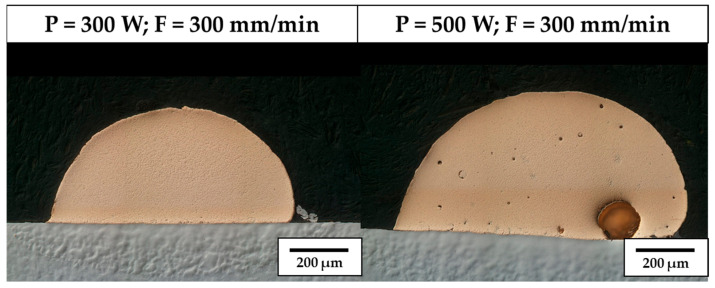
Influence of P on the structural integrity of CuSn_11_Bi_3_ STs.

**Figure 6:**
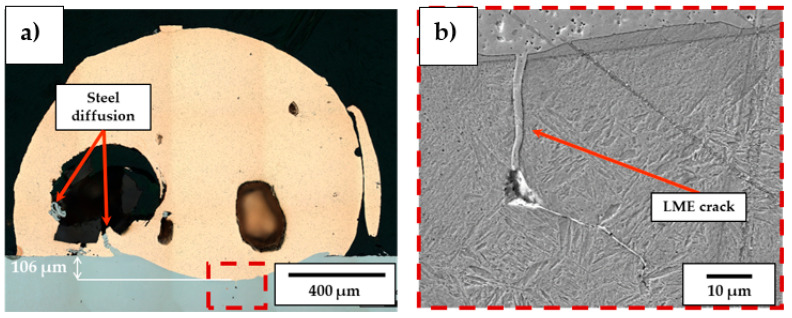
Steel diffusion on CuSn_11_Bi_3_ clad (**a**) and LME crack formation (**b**) for P = 800 W, F = 300 mm/min.

**Figure 7:**
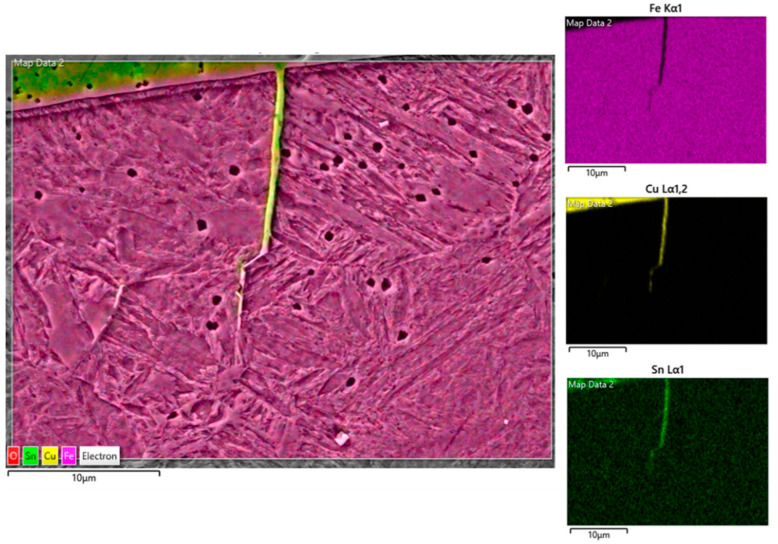
EDX mapping showing LME cracks filled by Cu and Sn.

**Figure 8:**
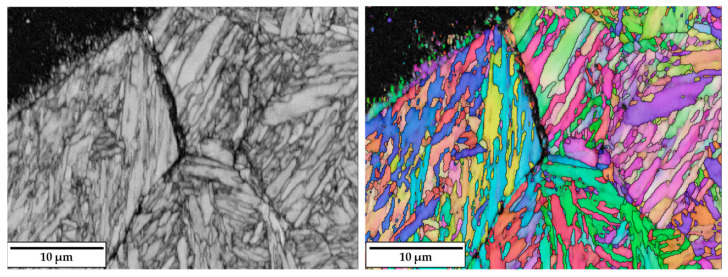
Band contrast image (**left**) and EBSD map (**right**) showing LME propagation along grain boundaries.

**Figure 9:**
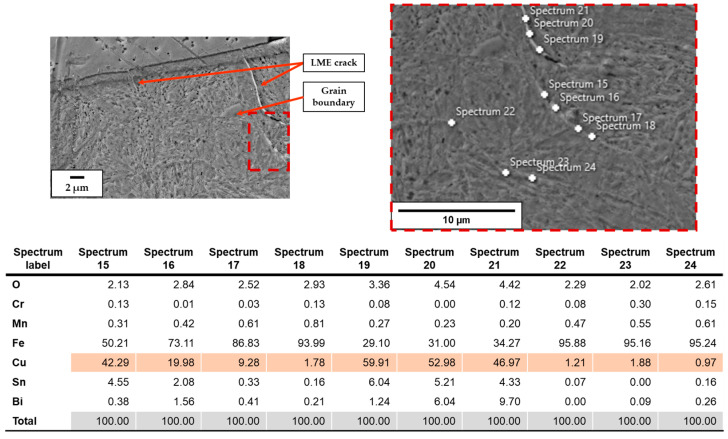
EDX point analysis performed on an LME crack and its surrounding area.

**Figure 10:**
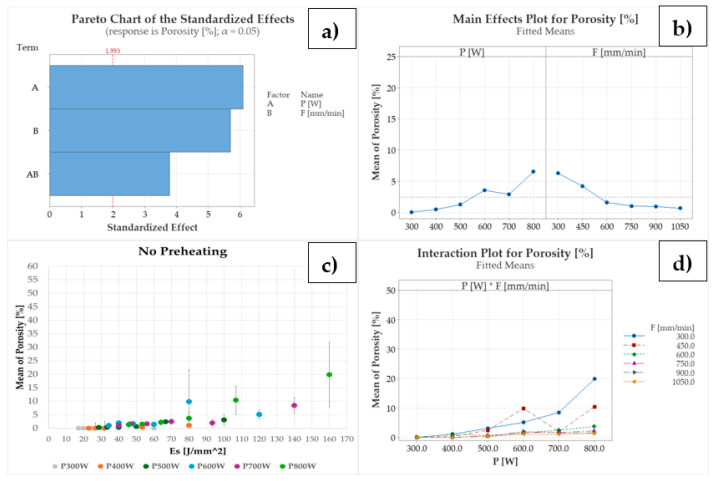
Influence of P, F, and E_s_ on the observed ST clad internal porosity for no substrate preheating: (**a**) Pareto chart, (**b**) P and F main effect plots, (**c**) ST clad internal porosity vs. E_s_., (**d**) P and F interaction plot.

**Figure 11:**
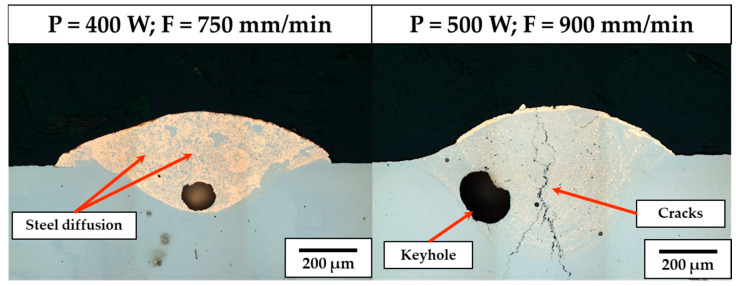
345 °C substrate preheating: Steel diffusion into the Cu-based clad (**left**), keyhole and crack formation (**right**).

**Figure 12:**
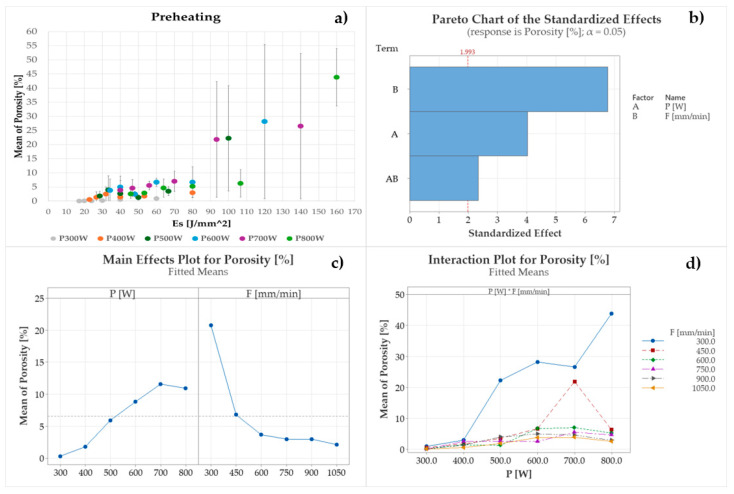
Influence of P, F, and E_s_ on the observed ST clad internal porosity for 345 °C substrate preheating: (**a**) ST clad internal porosity vs. E_s_., (**b**) Pareto chart, (**c**) P and F main effect plots, (**d**) P and F interaction plot.

**Figure 13:**
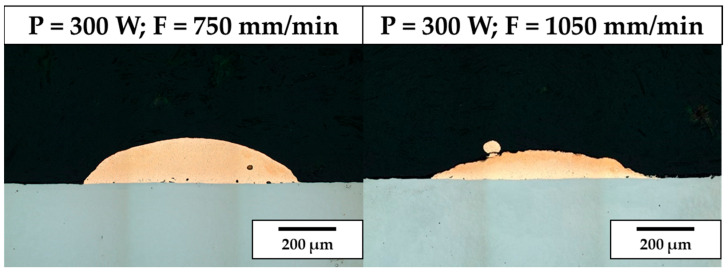
Substrate preheating of 345 °C: crack-free CuSn_11_Bi_3_ STs at 300 W.

**Figure 14:**
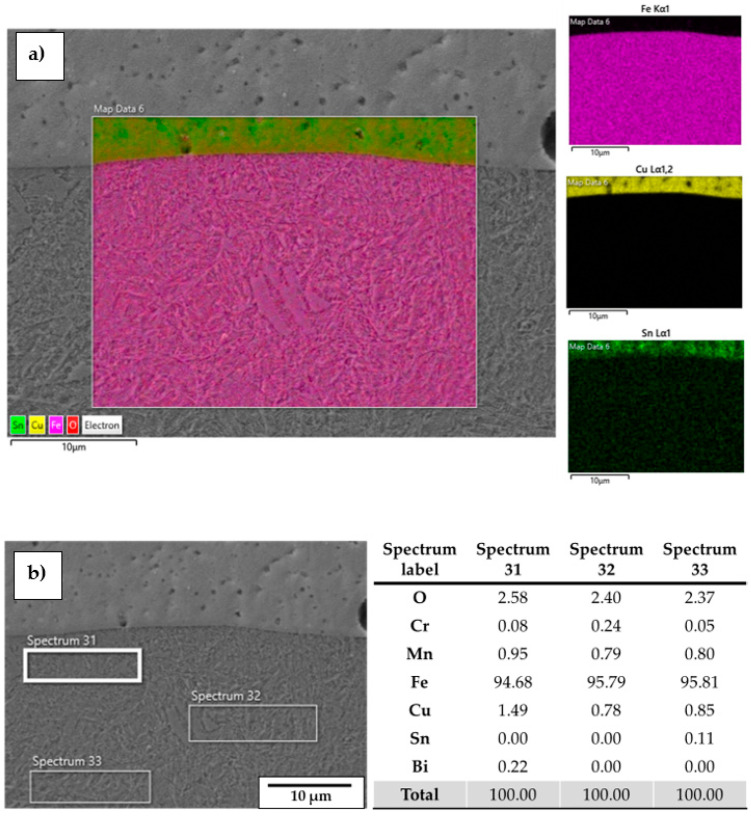
ST cross-section realized at P = 300 W and F = 750 mm/min: (**a**) EDX map; (**b**) EDX point analysis.

The performed SEM optical analysis demonstrates that the influence of the three considered LMD input variables on the structural integrity of CuSn_11_Bi_3_ STs realized on the C45 substrate is significant (see Figure 5, Figure 6, Figure 7, Figure 8, Figure 9, Figure 10, Figure 11, Figure 12, Figure 13 and Figure 14). To properly evaluate the effect of the LMD process parameters on the final quality of the as-deposited Cu-based material, the specific energy, E_s_ [J/mm^2^], the linear mass density, ρ [g/mm], and the net laser power reaching the C45 substrate, P_net_ [W], must be introduced. E_s_ is defined as:

(1) $$E_{s}=\frac{P}{F\times d}$$

where d is the laser spot diameter (see Table 3). E_s_ is a common LMD process parameter used to describe the energy balance during the deposition process, and it is directly proportional to laser power and inversely proportional to axis speed and laser spot. The linear mass density provides a measure of the melt pool powder supply, and it is defined as:

(2) $$\varrho =\frac{f}{F}$$

where f [g/s] is the powder feeding rate. Finally, P_net_ is the amount of laser power that reaches the metallic substrate net of energy losses due to heat powder absorption (P_powder_) and laser scattering and reflection (P_loss_) [50]. It is defined as:

(3) $$P_{net}=P_{nom\;}-\;P_{powder}-\;P_{loss}$$

where P_nom_ is the nominal laser power provided during the deposition process.

When no substrate preheating is involved, no melt pool penetration depth is observed for P ranging between 300 W and 600 W (see Figure 5), meaning that most of the P_nom_ is reflected and scattered by the powder flow exiting from the deposition nozzles. As a result, the P_net_ reaching the substrate is too low for melt pool formation, and the metal powder particles melt while still in suspension, dropping onto the C45 surface without forming any dilution zone. The observed phenomenon is in agreement with [51], where energy dispersion due to scattering was demonstrated to reach up to 97% of P_nom_ for the LMD of highly reflective spherical-shaped metal powder. By increasing P, substrate melting occurs, and melt pool penetration depths up to 106 µm are observed (see Figure 6a). The increased E_s_ promotes the formation of dilution zones and the mixing between CuSn_11_Bi_3_ and C45, inducing the nucleation of LME cracks, which propagate into the steel substrate (see Figure 6b). As discussed in [43], liquation cracks take place due to the diffusion of molten Cu into the steel grain boundaries, which deteriorates the cohesion between grains and entails a reduction in the minimum stress for intergranular cracking when high thermal loads are applied. Figure 7 and Figure 8 show LME cracking detected on the ST cross-section realized with 800 W and 300 mm/min. At high P levels, the EDX map reveals Cu and Sn diffusion within the observed crack (see Figure 7), which propagates into the C45 substrate following grain boundary directions, as demonstrated by the EBSD map in Figure 8.

The diffusion of Cu into the steel substrate is also confirmed by the EDX point analysis when a medium-high P is used (i.e., 600 W and 700W), which demonstrates the presence of copper along C45 grain boundaries even when no LME cracks are observed on spectra 22, 23, and 24 of Figure 9.

The influence of P and F on the clad internal porosity is significant, as highlighted by the Pareto chart (see Figure 10a). By moving from 300 W to 800 W, internal porosity increases from 0.03% up to 6.50% on average (see Figure 10b), detecting pores with a maximum size of 460 µm (see Figure 6). The strong increase in the internal porosity with P values ranging from 600 W to 800 W is explained by the low melting point and the high coefficient of thermal conductivity of the involved bronze alloy (see Table 2). Indeed, assuming P_loss_ as a function of particle shape and optical material properties, for constant axis speeds, an increase in E_s_ induces an increment in the laser energy absorbed by the powder particles as they overheat and evaporate, forming large gaseous cavities. The high cooling rates of the Cu-based material do not allow the vaporized metal to escape from the melt pool, trapping the gas in the clad cross-section and forming keyholes, which deteriorate the structural integrity of the as-built material.

The pronounced impact of P on the internal porosity of the as-deposited clad and on keyhole formation is further highlighted by Figure 10c. For constant E_s_ (i.e., 40 J/mm^2^, 60 J/mm^2^, and 80 J/mm^2^), an increase in laser power results in a deterioration of internal density, causing a strong keyhole formation for Es equal to or exceeding 80 J/mm^2^, as confirmed by [48,49] and demonstrated by the high data variability shown in Figure 10c. Indeed, keyhole is an unstable phenomenon that causes the formation of large and irregular pores that significantly affect porosity measurement. As discussed in Section 2.2, having performed a discrete measurement of the internal density of the as-deposited clad based on EDM cuts and optical imaging, the same repetitions of process parameters can exhibit very different internal porosities depending on the presence or absence of keyholes. This fact leads to a wide variability in the observed results for those combinations of process parameters that involve medium-high laser power.

Contrary to P, clad internal density increases with F (see Figure 10b,d). Moving from 300 mm/min to 1050 mm/min, internal porosity decreases below 1.5% on average. Moreover, by decreasing E_s_ through an increase in axis speed (i.e., F ranging between 750 mm/min and 1050 mm/min), no keyholes are observed. Nevertheless, for 300 W, lack of deposition occurs for ρ ranging between 7 × 10^−3^ g/mm and 9 × 10^−3^ g/mm as a result of the low specific energy and reduced melt pool powder supply induced by the high LMD axis speeds, degrading the structural integrity of the as-built clad.

In the case of no substrate preheating, high-quality and crack-free CuSn_11_Bi_3_ STs with an internal density of 99.97% are obtained using 300 W in combination with F ranging between 300 mm/min and 600 mm/min, and 400 W in combination with F ranging between 750 mm/min and 1050 mm/min (see Table 4).

The use of 345 °C of substrate preheating promotes large melt pool penetration depths also for medium-low P levels, causing extensive steel diffusion into the as-built CuSn_11_Bi_3_ clad for P ranging between 400 W and 800 W (see Figure 11, left). As previously discussed, the presence of dilution zones induces the nucleation of liquidation cracks at the CuSn_11_Bi_3_-C45 interface, which, in this case, propagate along the clad cross-sections, significantly deteriorating the structural integrity of the bimetallic deposition (see Figure 11, right). The propagation of LME cracks is due to the diffusion of steel into the bronze alloy and the resulting reduced ductility of the as-built material, which breaks due to the residual stresses formed by volumetric shrinkage during melt pool solidification and the different thermal expansion coefficients between the substrate and the dilution zone during cooling. In contrast to the case of no substrate preheating, keyhole phenomena are also observed for 400 W and axis speeds up to 900 mm/min, as confirmed by the presence of potato-shaped pores with a size ranging between 80 µm and 270 µm (see Figure 11).

These findings indicate that substrate preheating promotes keyhole formation, extending the deterioration of internal clad integrity even at E_s_ below 80 J/mm^2^. Notably, as shown in Figure 12a, a high result variability exists even at E_s_ equal to or exceeding 32 J/mm^2^ when 400 W is involved. Although P and F are still significant in affecting pore formation and extension, for 345 °C of substrate preheating, the effect of axis speed is predominant on laser power, as demonstrated by the Pareto chart in Figure 12b. By increasing F up to 1050 mm/min, the formation of gas inclusions due to Cu vaporization is reduced, although not prevented, observing a reduction of internal porosity from 20.76% to 2.09% on average (see Figure 12c,d).

High-quality ST depositions are achieved for laser power of 300 W and axis speeds ranging between 600 mm/min and 1050 mm/min (see Figure 13 and Table 4). EDX chemical analysis confirms that for the LMD combinations of process parameters in which no melt pool penetration depth occurs, no Cu diffusion into the C45 substrate is observed (see Figure 14a), preventing LME crack formation and preserving the starting chemical composition of the raw metal powder. The small amount of Cu observed in Figure 14b is attributed to the secondary electron bounce back of the CuSn_11_Bi_3_ layer since a negligible presence of Sn and Bi is detected.

### 3.2. CuSn_11_Bi_3_ Coating Analysis

The CLs produced using the LMD parameter combinations listed in Table 4 exhibited no LME cracks at the CuSn_11_Bi_3_-C45 interface, achieving final actual heights ranging between 0.162 mm and 0.530 mm. Cross-section analysis reveals internal densities higher than 99.00%, achieving a minimum internal porosity when an inert deposition environment is implemented in conjunction with 300 W, 750 mm/min, and 345 °C of substrate preheating (see Table 5).

**Figure 15:**
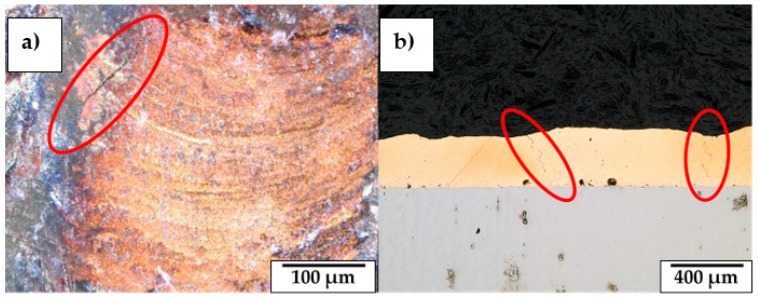
Structural cracks observed on CuSn_11_Bi_3_ CL realized at P300W, F900mm/min: (**a**) external surface; (**b**) cross-section.

**Figure 16:**
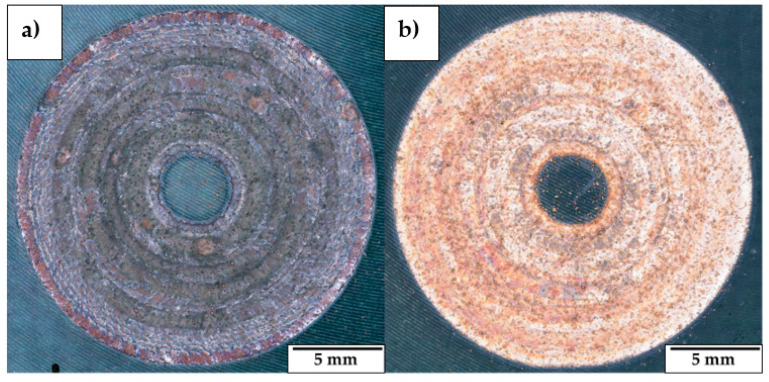
CuSn_11_Bi_3_ CL realized with P 300 W, F 900 mm/min, and 345 °C preheat: (**a**) 21% O_2_ content; (**b**) 0.02% O_2_ content.

**Figure 17:**
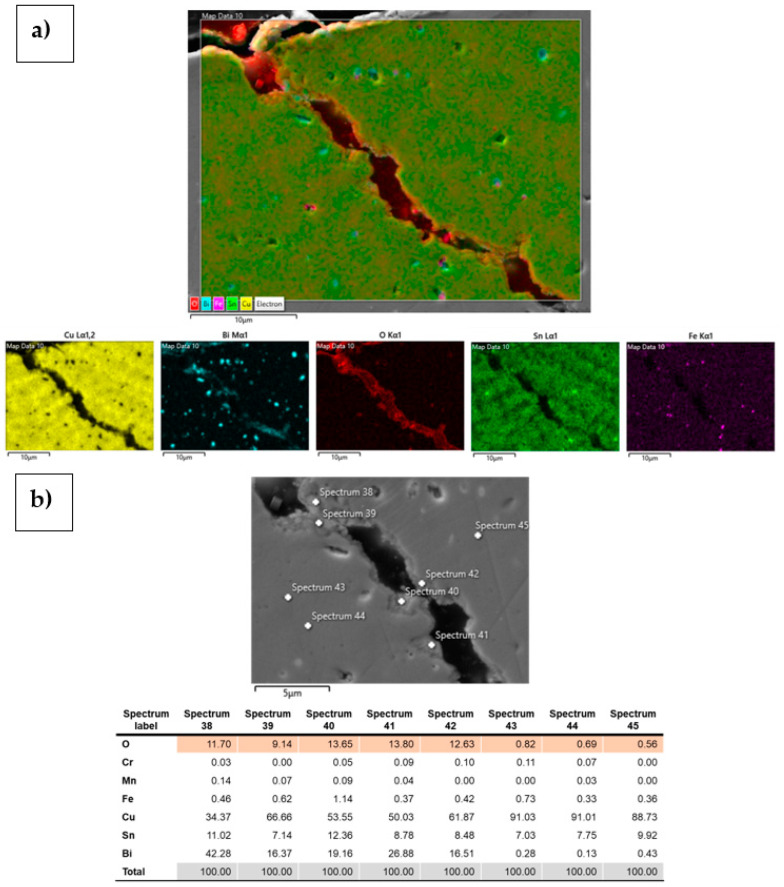
Structural cracks observed in the CL cross-section realized at P 300 W and F 750 mm/min in an open deposition environment: (**a**) EDX map; (**b**) EDX point analysis.

Nevertheless, structural cracks are observed in the Cu-based clad when CLs are realized in an open deposition chamber (see Table 6 and Figure 15a). The detected cracks initiate from the external surface of the realized sample and propagate into the bulk, degrading the structural integrity of the as-built material (see Figure 15b). On the contrary, samples realized with an enclosed deposition environment exhibit crack-free cross-sections, meaning that the reduction of O_2_ content in the LMD process improves the structural integrity of CuSn_11_Bi_3_ coatings. The reason for sample degradation is related to the high chemical reactivity of Cu and Bi with O_2_ at high temperatures. When LMD is performed in an open deposition chamber, the molten bronze alloy reacts with O_2_, forming a surface oxide layer that, due to its brittle mechanical behavior, is inclined to break when subjected to the thermal cycles, promoting crack formation and propagation. On the contrary, by limiting the content of O_2_ below 0.02%, oxidation is reduced, and crack formation is prevented. Figure 16 compares a coating realized with and without the use of an enclosed deposition environment, whereas Figure 17 shows both the EDX map and EDX point analysis performed on the structural cracks observed in CL realized at P 300 W, F 750 mm/min, and an open deposition environment. Chemical analysis reveals a high presence of O inside the observed crack and along the crack edges, confirming that significant oxidation forms when the Cu-based coating is realized with 21% O_2_ content (see Figure 16a). On the contrary, for an O_2_ content < 0.02%, superficial oxidation is strongly reduced, and the realized coating appears glossy (see Figure 16b).

## 4. Conclusions

**Figure 18:**
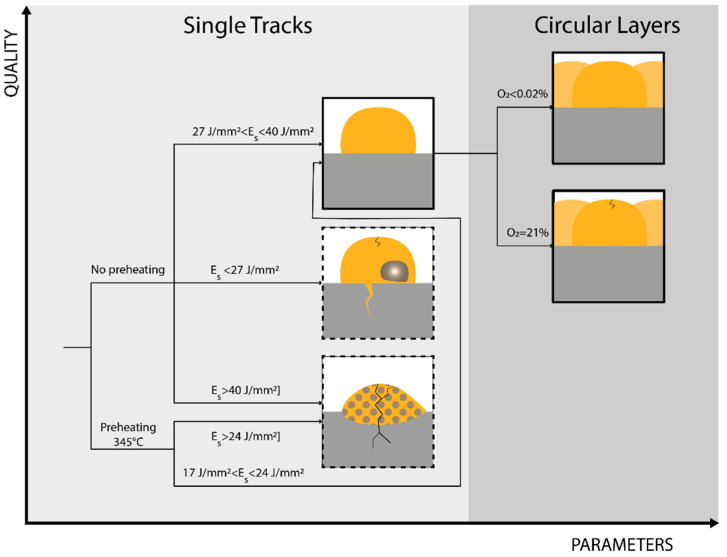
Diagram showing the evolution of the observed internal defects with regard to the involved Es and substrate preheating.

In this research work, the influence of the LMD process parameters on the manufacturing of high-density, crack-free CuSn_11_Bi_3_ depositions on C45 surfaces is analyzed by characterizing the influence of laser power, axis speed, substrate preheating, ST overlapping, and environmental printing conditions in terms of O_2_ percentage inside the building chamber on the final structural integrity of single circular layers. Figure 18 summarizes the evolution of the observed internal defects in relation to the levels of E_s_ and substrate preheating involved in the experimental campaign. The main experimental outputs highlight:

- High-density (>99%) and crack-free CuSn_11_Bi_3_ clads on C45 are achieved through specific energy ranging between 17 J/mm^2^ and 40 J/mm^2^;
- The employment of high energy densities (E_s_ > 40 J/mm^2^) degrades the structural integrity of the as-deposited material, inducing keyholes and LME cracks as a result of Cu diffusion along steel grain boundaries;
- The use of high laser power (ranging from 500 W to 800 W) and of substrate preheating produces large dilution zones and steel diffusion into the Cu-based clad, promoting brittle mechanical behavior of the as-deposited material and the propagation of structural cracks into as-built ST cross-sections;
- In crack-free depositions, no Cu diffusion into the C45 substrate is observed;
- In CL fabrication, no delamination is detected regardless of the use of substrate preheating;
- In Cu-based LMD, an enclosed deposition environment improves the final quality of the as-built coating by inhibiting material oxidation and preventing the formation of internal cracks that degrade the structural integrity of the deposition;
- Based on the achieved results, LMD demonstrates the capability of realizing thicker high-quality coatings compared to electroplating, ranging from 0.162 mm to 0.530 mm, which makes this AM technology more efficient in manufacturing bimetallic parts for the hydraulic, power generation, and aerospace sectors;
- In addition, despite Thermal Spraying (TS) being a competing technology for coating manufacturing, the high susceptibility of Cu-based alloys to form oxides and the moderate porosity typically featured by TS make process optimization challenging for the manufacture of defect-free Cu-Steel bimetallic components. On the contrary, the possibility of implementing inert deposition chambers and internal densities higher than 99% increases LMD attractiveness at the industrial level.

## 5. Future Works

Based on the main experimental findings, the next research activities will be related to (a) the characterization of the tribological properties of the as-built CL coating and (b) the optimization of the LMD process for the manufacturing of CuSn_11_Bi_3_ multi-layer depositions to enable thicker Cu-based depositions. Indeed, steel components coated with Cu-based materials generally exhibit a coating thickness exceeding 1 mm; therefore, it is essential to achieve coatings with a greater thickness than those measured on the CLs. To address this limitation, future efforts should focus on investigating how the alteration of the chemical and optical properties of the deposited material—resulting from the prior deposition of copper-based layers—affects the performance of the LMD process and the final coating quality. Structural characterizations, including porosity analysis, mechanical testing, and residual stress measurements, will be performed, and the follow-up activities will be extended to industrial use cases.

## Figures and Tables

**Table 1:** CuSn_11_Bi_3_ and C45 chemical composition (wt. %).

Material	Chemical Composition							
CuSn_11_Bi_3_	Bi: 3.00		Sn: 11.00		Ni: 2.00 max		Cu: Bal.	
C45	C: 0.45	Si: 0.40		Cr: 0.40		Ni: 0.40		Fe: Bal.

**Table 2:** CuSn_11_Bi_3_ and C45 main thermo-mechanical properties.

Material	Bulk Density at 20° C [g/cm^3^]	Yield Strength [MPa]	Young Modulus [GPa]	Thermal Expansion Coefficient [10^−6^ °C^−1^]	Solidus/Liquidus Temperature [°C]
CuSn_11_Bi_3_	8.94	220	105	18	778/985
C45	7.8	300	210	12	1460/1420

**Table 3:** LMD process parameters involved in ST depositions.

LMD Process Parameter	Min. Value	Max. Value	Number of Levels	Step Value
Laser power [W]	300	800	6	100
Axis speed [mm/min]	300	1050	6	150
Preheating [°C]	20	345	2	-
E_s_ [J/mm^2^]	17	160	-	-
ϱ [g/mm]	7 × 10^−3^	23 × 10^−3^	-	-
Laser spot [mm]	1		-	-
Powder feed [g/s]	0.116		-	-
Stand-off [mm]	10.5		-	-
Carrier [l/min]	4		-	-
Shielding [l/min]	16		-	-

**Table 4:** LMD process parameters for CL printing.

P [W]	F [mm/min]	Preheating [°C]	O_2_ Content [%]	E_s_ [J/mm^2^]	ρ [g/mm]
300	450	-	0.02/21	40	0.015
400	750	-	0.02/21	32	0.009
400	900	-	0.02/21	27	0.008
300	750	345	0.02/21	24	0.009
300	900	345	0.02/21	20	0.008
300	1050	345	0.02/21	17	0.007

**Table 5:** CuSn_11_Bi_3_ CLs: detected internal porosity and actual height.

P [W]	F [mm/min]	ST Overlap [%]	Preheating [° C]	Internal Porosity [%]		CL Actual Height [mm]	
				O_2_ = 21%	O_2_ < 0.02%	O_2_ = 21%	O_2_ < 0.02%
300	450	60	-	0.45	0.15	0.530	0.336
400	750	60	-	0.51	0.99	0.307	0.215
400	900	60	-	0.66	0.53	0.262	0.162
300	750	60	345	0.82	0.03	0.432	0.315
300	900	60	345	0.83	0.2	0.315	0.245
300	1050	60	345	0.67	0.67	0.246	0.190

**Table 6:** Structural cracks observed in the realized CuSn_11_Bi_3_ CLs.

P [W]	F [mm/min]	ST Overlap [%]	Preheating [°C]	Structural Cracks	
				O_2_ = 21%	O_2_ < 0.02%
300	450	60	-	Yes	No
400	750	60	-	No	No
400	900	60	-	No	No
300	750	60	345	Yes	No
300	900	60	345	Yes	No
300	1050	60	345	Yes	No

## Data Availability

The original contributions presented in this study are included in the article. Further inquiries can be directed to the corresponding author.
